# Increased breast cancer cell toxicity by palladination of the polyamine analogue *N*^1^,*N*^11^-bis(ethyl)norspermine

**DOI:** 10.1007/s00726-013-1621-y

**Published:** 2013-12-21

**Authors:** Tania M. Silva, Sonia M. Fiuza, Maria P. M. Marques, Lo Persson, Stina Oredsson

**Affiliations:** 1 Research Unit “Molecular Physical-Chemistry”, University of Coimbra, Coimbra, Portugal; 2 Department of Biology, University of Lund, Lund, Sweden; 3 Department of Experimental Medical Science, University of Lund, Lund, Sweden; 4 Department of Life Sciences, Faculty of Science and Technology, University of Coimbra, Coimbra, Portugal

**Keywords:** Breast cancer, Polyamine analogues, Palladium (Pd)(II) complexes, Platinum (Pt)(II) complexes, DNA damage, Cancer stem cells

## Abstract

Breast cancer is one of the most common malignant tumor forms among women and many women succumb to their disease. Thus, new anticancer agents that can efficiently improve patient survival are of the utmost importance. In this study, the effects of the polyamine analogues *N* ^1^,*N* ^11^-bis(ethyl)norspermine (BENSpm) and *N* ^1^-cyclo-propylmethyl-*N* ^11^-ethylnorspermine (CPENSpm) and the synthesized dinuclear complexes Pd_2_BENSpm (Pd-BENSpm), Pt_2_CPENSpm (Pt-CPENSpm) and Pd_2_Spm (Pd-Spm) were investigated in normal-like breast epithelial MCF-10A cells and the breast cancer cell lines JIMT-1 and L56BR-C1. The overall data show that palladination of BENSpm resulted in enhanced cytotoxicity, in contrast to platination of CPENSpm that reduced cytotoxicity, which might be explained by differences in the cellular uptake of Pd-BENSpm and Pt-CPENSpm. BENSpm and Pd-BENSpm treatment reduced the CD44^+^CD24^−^ putative cancer stem cell population, evaluated by flow cytometry. Furthermore, Pd-BENSpm was the most efficient compound regarding induction of DNA damage and decrease in colony formation in soft agar. Pt-CPENSpm and Pd-Spm, on the other hand, were shown to be the least toxic compounds of all tested. Pd-Spm efficiently reduced the cellular glutathione levels, which probably was a consequence of its metabolic inactivation by conjugation to this endogenous thiol. The normal-like cells were found to be less sensitive to the agents than the breast cancer cells. Our findings show that Pd-BENSpm exhibits promising anticancer effects which render it suitable for further optimization to develop a new metal-based chemotherapeutic drug for breast cancer treatment.

**Electronic supplementary material:**

The online version of this article (doi:10.1007/s00726-013-1621-y) contains supplementary material, which is available to authorized users.

## Introduction

The antiproliferative features of cisplatin, [*cis*-diamminedichloroplatinum (II)], (*cis*-Pt(NH_3_)_2_Cl_2_), were discovered in 1965 by Barnett Rosenberg and resulted in the successful use of this compound, as the first metal complex, in the treatment of a wide range of solid tumors (Rosenberg et al. 1965). Platinum (Pt)-based agents are widely used as chemotherapeutic compounds in today’s oncological practice, based on their ability to enter the cell nucleus and covalently bind to DNA, yielding stable adducts (Brabec and Kasparkova 2005; Esteban-Fernandez et al. 2010). The formation of several cross-links with DNA leads to a distortion of the DNA molecule. Thus, essential biological processes, such as replication and transcription of DNA are inhibited, which affects protein synthesis and, consequently, cell proliferation (Brabec and Kasparkova 2005; Esteban-Fernandez et al. 2010). Problems with off-target effects such as neurotoxicity and nephrotoxicity, and the development of acquired resistance, are the main limiting factors of cisplatin treatment (Brabec and Kasparkova 2005; Esteban-Fernandez et al. 2010). In the few last years, palladium (Pd)(II) complexes have shown significant antitumor activity against different cancer cell lines, along with fewer off-target effects, as compared to cisplatin (Ulukaya et al. 2011).

Another group of compounds extensively used in experimental cancer research are polyamine analogues (Casero and Woster 2009). The natural polyamines putrescine, spermidine and spermine are a group of ubiquitous positively charged substances with low molecular weight (Wallace et al. 2003; Palmer and Wallace 2010). In all eukaryotic cells, the polyamines are involved in a large number of fundamental biological processes, such as in the regulation of cell proliferation, differentiation and death (Wallace et al. 2003; Palmer and Wallace 2010; Traquete et al. 2013). Although all of the exact roles of polyamines in these processes are not known, the capacity to interact with DNA and affect DNA conformation are thought to play a role in their normal cellular function (Iacomino and Picariello 1823; Pasini et al. 2013). Polyamine pools are in general higher in malignant tissue than in normal tissue (Pegg 1988; Traquete et al. 2013), which has suggested the polyamine metabolic pathway as a target for anticancer therapy (Pegg 1988; Seiler 2005; Casero and Woster 2009; Palmer and Wallace 2010). Polyamine pool depletion results in inhibition of cell proliferation and, sometimes, in induction of cell death. One means to achieve polyamine depletion is to treat with polyamine analogues, which stimulate polyamine catabolism and inhibit polyamine biosynthesis, while they cannot functionally substitute for the depleted biogenic polyamines (Davidson et al. 1999; Oredsson et al. 2007; Casero and Woster 2009). Among the most promising studied polyamine-based antitumor compounds are the *N*-ethyl-substituted polyamines, such as *N* ^1^,*N* ^11^-bis(ethyl)norspermine (BENSpm), a symmetrically substituted spermine analogue and *N* ^1^-cyclo-propylmethyl-*N* ^11^-ethylnorspermine (CPENSpm), an unsymmetrically substituted spermine analogue. They have been found to down-regulate polyamine biosynthesis, up-regulate catabolism and compete for polyamine uptake, resulting in dramatic polyamine depletion, inhibition of cell proliferation and, sometimes, induction of apoptosis (Davidson et al. 1999; Casero and Woster 2009).

A way to increase the efficiency of a chemotherapeutic agent, *e.g.* by increasing interaction with DNA, may be to combine two different compounds for which DNA is the biological target, for instance Pt(II) and Pd(II) complexes with polyamine ligands. In fact, linear alkylpolyamines are suitable ligands for both Pt(II) and Pd(II) ions, and yield polynuclear chelates capable of disrupting the native DNA conformation through the formation of non-conventional (long-range, interstrand) interactions with the N_7_ atom of the purine bases (Hegmans et al. 2008; Ulukaya et al. 2011). Several promising results with these complexes have been obtained over the last years (Lebwohl and Canetta 1998; Marques et al. 2002; Fiuza et al. 2006; Fiuza et al. 2011; Miklasova et al. 2012; Silva et al. 2012).

We have shown that several breast cancer cell lines are highly sensitive to treatment with the Pd(II) chelate of the polyamine analogue norspermidine (NSpd) and that this chelate was more toxic than its Pt(II) counterpart (Silva et al. 2013). Also, a palladinated spermine was found to be cytotoxic against breast cancer cell lines (Fiuza et al. 2011).

In the present study, we investigate the cytotoxic effects of several Pd(II) and Pt(II) polyamine complexes against two human breast cancer cell lines (JIMT-1 and L56Br-C1) and one immortalized normal-like breast epithelial cell line (MCF-10A): two newly synthesized Pd(II) and Pt(II) chelates Pd_2_BENSpm (Pd-BENSpm) and Pt_2_CPENSpm (Pt-CPENSpm) (Silva et al. 2012)—and the complex Pd_2_Spm (Pd-Spm). Altogether, the results show that palladination of BENSpm resulted in an increased cytoxicity relative to the other tested compounds.

## Materials and methods

### Chemicals

Cell culture medium components were purchased from Biochrom, Berlin, Germany. Tissue culture plastics were acquired from Nunc, Roskilde, Denmark. Phosphate-buffered saline (PBS: 8 g/L NaCl, 0.2 g/L KCl, 1.15 g/L Na_2_HPO_4_, 0.2 g/L KH_2_PO_4_, pH 7.3) was purchased from Oxoid Ltd., Basingstoke, Hampshire, UK. Nonidet P-40 was purchased from VWR, Lund, Sweden. Insulin, hydrocortisone, propidium iodide (PI), Accutase, 3-(4,5-dimethyl-thiazolyl-2)-2,5 diphenyltetrazolium bromide (MTT) and poly(2-hydroxyethyl methacrylate) (polyHEMA) were obtained from Sigma, Stockholm, Sweden. Epithelial growth factor was purchased from Invitrogen AB, Stockholm, Sweden. Dimethyl sulphoxide (DMSO) was acquired from Merck KGaA, Darmstadt, Germany. ^14^[C]Acetyl-coenzyme A was purchased from New England Nuclear, DuPont, Scandinavia AB, Stockholm, Sweden. The monoclonal antibodies CD44-fluorescein isothiocyanate (FITC) and CD24-phycoerythrin (PE) together with the FITC- and PE-conjugated mouse IgG1 isotype controls were obtained from Becton–Dickinson, Stockholm, Sweden. Nusieve^®^ GTG low-melting-point agarose, agarose gel supporting medium and Gel Bond^®^ membranes were obtained from FMC BioProducts, Rockland, ME, USA. The GSH-Glo™ Glutathione (GSH) kit was purchased from Promega Biotech AB, Nacka, Sweden. The Pd-Spm complex was synthesized by Dr. Sónia Fiuza (Fiuza et al. 2011). BENSpm and CPENSpm were synthesized and kindly provided by Dr. Patrick Woster, Department of Drug Discovery and Biomedical Sciences, Medical University of South Carolina, USA (Casero and Woster 2009). Pd-BENSpm and Pt-CPENSpm complexes were synthesized as previously described (Silva et al. 2012). The complexes are fully characterized by elemental analysis, as well as through vibrational spectroscopy (Raman and FTIR). The purity of the analyzed compounds is therefore assured (Silva et al. 2012).

### Drug stock solutions

Stock solutions (2 mM) of BENSpm and CPENSpm were made in PBS, sterile-filtered and stored at 4 °C. Pd-BENSpm and Pd-Spm were dissolved in 4 % DMSO in PBS to give stock solutions of 1 mM that were sterile-filtered and stored at −20 °C. Pt-CPENSpm was dissolved in 4 % DMSO in PBS to give a stock solution of 2 mM, sterile-filtered and stored at −20 °C. Further dilutions were made in complete cell culture medium to give the final concentrations.

### Cell lines and cell culturing

The L56Br-C1 cell line was established at the Department of Oncology, Clinical Sciences, Lund University, Sweden (Johannsson et al. 2003). The JIMT-1 cell line was purchased from the German Collection of Microorganisms and Cell Cultures (Braunschweig, Germany) and the MCF-10A cell line was obtained from the American Tissue Type Culture Collection (Manassas, VA, USA). The cell lines were cultured as previously described (Silva et al. 2013). For all experiments, the cells were seeded and allowed to attach and grow for 24 h, before addition of compound at a 10 μM concentration. A concentration range between 0.1 and 100 μM was used in the MTT assay. The control received DMSO at the same final concentration as that in the treated cultures, i.e., 0.1–0.2 %.

### Dose response assay

The MTT assay was performed as previously described (Holst and Oredsson 2005). Briefly, cells were seeded in 96-well microplates with a seeding density of 3,000 (MCF-10A), 5,000 (JIMT-1) or 8,000 (L56Br-C1) cells in 180 μl of medium. At 24, 48 and 72 h of drug treatment, 20 μl of MTT solution (5 mg/ml MTT in PBS) was added to the cells, which were incubated for 1 h at 37 °C. After removal of the MTT containing medium, the cells containing insoluble formazan crystals were dissolved by addition of 100 μl of 100 % DMSO per well. Absorbance was monitored at 540 nm in a Labsystems iEMS Reader MF (Labsystems Oy, Helsinki, Finland) using the DeltaSoft II v.4.14 software (Biometallics Inc., Princeton, NJ, USA).

### Cell proliferation

Cells were seeded in Petri dishes (5 cm diameter) at a density of 0.3 × 10^6^ cells/Petri dish (MCF-10A cells) or 0.6 × 10^6^ cells/Petri dish (JIMT-1 and L56Br-C1 cells) in 5 ml of medium. The cells were allowed to attach and grow for 24 h and were further harvested by trypsinization, with the cell number being determined by counting in a hemocytometer after 24, 48 and 72 h of treatment.

### Effect of long-time exposure on cell proliferation

This experiment was design to investigate the effect of the tested compounds on cancer cell proliferation over a longer period and was performed as previously described (Silva et al. 2013). Cells (0.3 × 10^6^ MCF-10A cells, 0.7 × 10^6^ JIMT-1 cells and 0.7 × 10^6^ L56Br-C1 cells) were seeded into 5 ml of medium in 25 cm^2^ cell culture flasks and the compounds were added to the final concentration of 10 μM 24 h later. At each passage, cells were harvested by trypsinization, counted in a hemocytometer, and reseeded at the same density as above. The cells received the same treatment during each passage. Each week of treatment is defined as a treatment cycle and the cells were subjected to five treatment cycles. The total recovery time, i.e., incubation without compound, was 96 h. The data are presented as the total amount of cells that theoretically would have accumulated if all cells had been reseeded with a known cell density after each treatment cycle. Thus, using the cell number obtained at each passage of a culture seeded with a known cell density, and applying the formula log *N* = log *N*_0_ + *n*log2, it was possible to calculate the number of cells that would have been obtained if all cells were reseeded at a lower density at each passage.

### Intracellular Pd(II) and Pt(II) accumulation

For the study of the intracellular accumulation of Pd-BENSpm, Pd-Spm or Pt-CPENSpm, cells (1 × 10^6^ MCF-10A cells and 2 × 10^6^ JIMT-1 and L56Br-C1 cells) were seeded into 12 ml of medium in Petri dishes (9 cm diameter). After 72 h of treatment with a 10 μM concentration of the tested compounds, cells were washed with ice-cold PBS, harvested by trypsinization, counted, pelleted by centrifugation and stored at −80 °C until analysis. Prior to analysis, the pellets were digested in 65 % HNO_3_ for 2 h at 65 °C, diluted to a 5 % acid solution and centrifuged at 600*g* for 14 min. The Pd(II) and Pt(II) accumulation was then analyzed by inductively-coupled plasma mass spectrometry (ICP-MS) (Thermo X7, Thermo Elemental, Winsford, UK). The data of metal content were used to calculate the intracellular concentration of the compounds.

### Analysis of polyamine content by high performance liquid chromatography

L56Br-C1 cells were seeded as described for the proliferation assay and, after 24 h of treatment, cells were harvested, counted, pelleted and stored at −20 °C until analysis. Chromatographic separation and quantitative determination of the polyamines in cell extracts in 0.2 M perchloric acid were carried out using high performance liquid chromatography (Hewlett Packard 1100) with *θ*-phthaldialdehyde as the reagent (Seiler and Knodgen 1985).

### Spermidine/spermine *N*^1^-acetyltransferase activity assay

L56Br-C1 cells were seeded as described for the proliferation assay and, after 24 h of treatment, cells were harvested, counted, pelleted and stored at −80 °C until analysis. The samples were sonicated in 50 mM Tris–HCl (pH 7.5) containing 0.25 M sucrose. The spermidine/spermine *N* ^1^-acetyltransferase (SSAT) activity was determined in the sonicates by measuring the synthesis of ^14^[C]acetylspermidine after incubation of the cell extracts with ^14^[C]acetyl-coenzyme A and spermidine, as previously described (Matsui et al. 1981).

### Cell cycle phase distribution and cell death analysis by flow cytometry

MCF-10A, JIMT-1 and L56Br-C1 cells were seeded as described for the proliferation assay and were further prepared for flow cytometric analysis, as previously described (Silva et al. 2013). The cells were analyzed using an Accuri C6 flow cytometer (BD Biosciences, San Jose, CA, USA). For the computerized evaluation of the cell cycle phase distribution and the sub-G_1_ region, the MultiCycle^®^ software program (Phoenix Flow Systems, CA, USA) was used. The sub-G_1_ region was evaluated as percentage signals in sub-G_1_ in relation to all the signals in the sub-G_1_, G_1_, S and G_2_ histograms. The distribution of the cells in G_1_, S and G_2_ phases was evaluated in % of all cells in G_1_, S and G_2_.

### Identification of the CD44^+^CD24^−^ putative cancer stem cell population by flow cytometry

JIMT-1 cells (0.6 × 10^6^ cells) were seeded into 5 ml of medium in Petri dishes (5 cm diameter) and, after a 72 h treatment period, cells were harvested by Accutase to prevent the disruption of the cell surface proteins. Afterward, the live cells were washed three times with 5 ml ice-cold PBS containing 1 % FCS and incubated while shaking for 15 min on ice with the monoclonal antibodies CD44-FITC and CD24-PE. Next, cells were again washed three times with ice-cold PBS containing 1 % FCS and identified based on their expression of the cell surface markers CD44 and CD24 using an Accuri C6 flow cytometer (BD Biosciences, San Jose, CA, USA). The data were analyzed using the CFlow software (BD Biosciences, San Jose, CA, USA).

### Colony formation in soft agar

JIMT-1 and L56Br-C1 cells were seeded and treated as described for the proliferation assay, before reseeding in soft agar at low density. After 72 h of treatment, cells were trypsinized, counted and resuspended in agarose containing medium (0.3 % agarose) at cloning density, i.e., one cell per microliter, and then reseeded (0.5 ml) in 48-well plates coated with polyHEMA (Cirenajwis et al. 2010). The cells were incubated at 37 °C in a humidified incubator with 5 % CO_2_ in air for 14 days and colonies were counted in an inverted phase contrast microscope.

### Single cell gel electrophoresis assay

The single cell gel electrophoresis (SCGE) assay was performed as previously described (Freiburghaus et al. 2012). Briefly, MCF-10A cells (0.3 × 10^6^ cells) and JIMT-1 and L56Br-C1 cells (0.7 × 10^6^ cells) were seeded into 5 ml of medium in Petri dishes (5 cm in diameter). After 72 h of treatment with a 10 μM concentration of the tested compounds, cells were harvested and special attention was given to the trypsinization time because trypsin can itself induce DNA damage. Consequently, the time of trypsinization was exactly 13 min for MCF-10A cells, 10 min for JIMT-1 cells and 6 min for L56Br-C1 cells.

The comets were investigated and photographed with an epifluorescence microscope (Olympus AX70 equipped with a Nikon DSRI1 camera). Seven to 22 random images were captured for each sample. In each image, all nucleoids were counted by visual inspection. Then, nucleoids with tails were visually counted in each image. A nucleoid with tail was defined as a nucleoid with a visible tail independent of tail size. The percentage of nucleoids with a tail in relation to all nucleoids was calculated. A mean was calculated for the counted nucleoids in all images of each sample. At least 30 nucleoids were scored for each sample. Afterwards, the mean of the samples for each treatment was calculated.

### GSH-Glo™ Glutathione assay

The GSH assay was performed according to the instructions of the manufacturer (GSH-Glo™ Glutathione Assay Technical Bulletin TB369). Briefly, cells were trypsinized, counted in a hemocytometer, pelleted and resuspended in cell culture medium. Aliquots of 180 μl cell suspension containing 3,000 (MCF-10A), 6,000 (JIMT-1) or 8,000 (L56Br-C1) cells were seeded in the wells of white, opaque flat-bottomed 96-well plates. At 48 h of drug treatment (10 μM), the medium was removed from the plates very carefully. Next, luciferin-NT and glutathione S-transferase were added to GSH-Glo™ reaction buffer to make GSH-Glo™ reagent, which was further added to the wells of the plates. Before a 30-min incubation period at room temperature, the plates were swirled gently and briefly. Then, the luciferin detection reagent was added to the wells of the plates, which were again gently and briefly swirled, followed by incubation for 15 min at room temperature. Finally, luminescence was monitored in a Labsystems iEMS Reader MF (Labsystems Oy, Helsinki, Finland) using the DeltaSoft II v.4.14 software (Biometallics Inc., Princeton, NJ, USA).

### Statistical analysis

For the statistical significance evaluation, a one-way ANOVA followed by the Newman–Keuls Multiple Comparison test was used. Differences were considered statistically significant at *p* < 0.05.

## Results

### MTT reduction

The reduction of MTT is assumed to be a measure of cell number (Holst and Oredsson 2005) and was initially used to access the toxicity of BENSpm, Pd-BENSpm, CPENSpm or Pt-CPENSpm against the three cell lines investigated, using concentrations that ranged from 0.1 to 100 μM and at 24, 48 and 72 h of treatment (Online Resource 1). The inhibitory concentration 50 (IC_50_) obtained after 72 h of treatment is shown in Table 1. The normal-like MCF-10A cells were least sensitive while the L56Br-C1 cell line was most sensitive. Palladination of BENSPM slightly increased cytotoxicity while platination of CPENSpm greatly decreased cytotoxicity. Based on these data and on previously published data on BENSpm and CPENSpm (McCloskey et al. 2000; Holst and Oredsson 2005; Oredsson et al. 2007; Uimari et al. 2009), we decided to use a 10 μM concentration for further studies.

**Table 1:** Absolute IC_50_ values (μM) of MCF-10A, JIMT-1 and L56-Br-C1 cells treated for 72 h with BENSpm, Pd-BENSpm, CPENSpm or Pt-CPENSpm

Cell line	MCF-10A	JIMT-1	L56Br-C1
BENSpm	52.8	8.7	0.4
Pd-BENSpm	34.2	7.3	0.4
CPENSpm	12.2	9.9	0.2
Pt-CPENSpm	>100 μM	>100 μM	>100 μM

Twenty-four hours after seeding of cells in 96-well plates, the compounds were added in a concentration range of 0.1–100 μM and the cells were treated for 72 h before evaluation using an MTT assay. The data were used to construct dose response curves for the evaluation of absolute IC_50_ (*n* = 12)

### Effect of one treatment cycle on cell proliferation

**Fig. 1:**
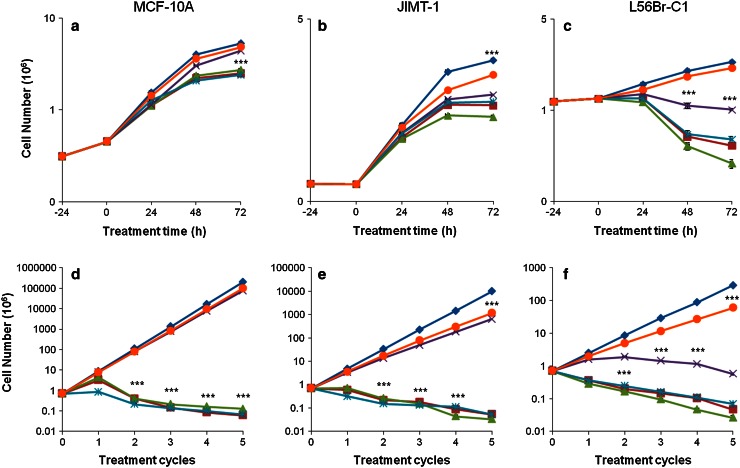
Effect of BENSpm, Pd-BENSpm, Pd-Spm, CPENSpm or Pt-CPENSpm treatment on cell proliferation. **a–c** One treatment cycle. Twenty-four hours after seeding of cells (0 h time of treatment in the figure), the compounds were added to a final concentration of 10 μM. Cells were harvested by trypsinization and counted in a hemocytometer. The results are presented as mean values (*n* = 6). SEM bars are covered by the symbols. **d**–**f** Five treatment cycles. Cells were seeded and the compounds were added to the final concentration of 10 μM, 24 h after seeding. After 72 h of treatment, the drug-containing medium was aspirated and drug free culture medium was added. After an additional 72 h of incubation, cells were harvested by trypsinization and counted in a hemocytometer. These 7 days were defined as one treatment cycle. The cells were reseeded at the same density as at the previous passage and treated with the same drug for the next treatment cycle. All together this was repeated for five treatment cycles. The total recovery time between repeated treatments was 96 h. The results are presented as mean values (*n* = 3). SEM bars are covered by the symbols. Please note that the y-axis has different scales for the different cell lines because of different rates of cell proliferation. ***p < 0.001 compared to control for the curves below the symbol.  Control,  BENSpm,  Pd-BENSpm,  Pd-Spm,  CPENSpm,  Pt-CPENSpm

We first investigated the effect of the compounds on cell proliferation during one treatment cycle (Fig. 1a–c). As also shown in the MTT assay, Pt-CPENSpm was the least cytotoxic compound. Actually, Pt-CPENSpm did not affect proliferation of MCF-10A (Fig. 1a) and L56Br-C1 (Fig. 1c) cells, but slightly inhibited proliferation of JIMT-1 (Fig. 1b) cells. Pd-Spm treatment did not affect proliferation of MCF-10A cells, while BENSpm, Pd-BENSpm or CPENSpm treatment slightly reduced the cell numbers at 48 and 72 h of treatment (Fig. 1a). In L56Br-C1 cells, treatment with BENSpm, Pd-BENSpm or CPENSpm resulted in a decrease in cell number after 48 and 72 h of treatment compared to the cell number at 24 h of treatment, implicating cell death (Fig. 1c). Pd-Spm also resulted in a slight decrease in cell number in L56Br-C1 cells. In JIMT-1 (Fig. 1b) cells, the inhibition of cell proliferation was similar after treatment with BENSpm, Pd-BENSpm, CPENSpm or Pd-Spm. In conclusion, the cell proliferation assay shows that the least sensitive cell line was MCF-10A and the most sensitive one was L56Br-C1.

### Effect of repeated treatment cycles on cell proliferation

In addition to investigating the effect after one 72 h treatment cycle, we also evaluated the effect of repeated treatment cycles with 10 μM BENSpm, Pd-BENSpm, Pd-Spm, CPENSpm or Pt-CPENSpm on cell proliferation of MCF-10A, JIMT-1 and L56Br-C1 cells (Fig. 1d–f). The cells were cultivated in cycles with drug treatment for 72 h followed by a treatment free period of 96 h in between, for a total number of five cycles. Proliferation of MCF-10A normal-like breast cells was not affected by repeated treatment cycles with either Pt-CPENSpm or Pd-Spm (Fig. 1d), while repeated treatments with these compounds had a somewhat inhibitory effect on the growth of JIMT-1 cells (Fig. 1e). In L56Br-C1 cells, repeated treatments with Pd-Spm resulted in a reduction of cell number after four treatment cycles (Fig. 1f). Repeated treatment with BENSpm, Pd-BENSpm or CPENSpm resulted in a decrease in the cell number, compared to the number of cells seeded at time 0, in all cell lines (Fig. 1d–f).

### Intracellular Pd(II) and Pt(II) accumulation by inductively-coupled plasma mass spectrometry

**Fig. 2:**
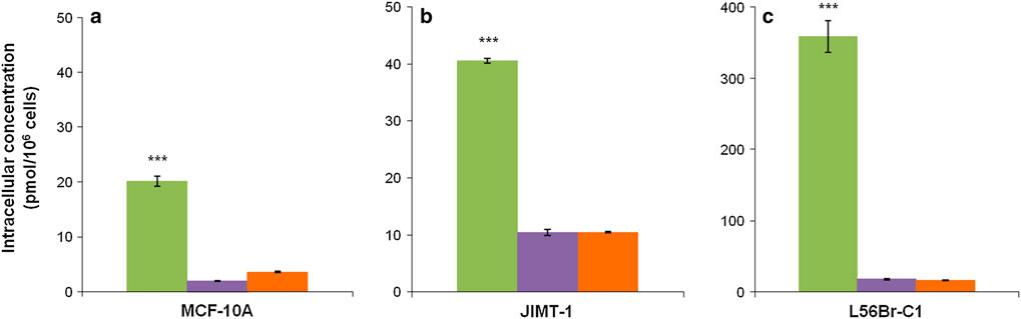
Intracellular concentration of Pd-BENSpm, Pd-Spm and Pt-CPENSpm in MCF-10A, JIMT-1 and L56Br-C1 cells. After 72 h of treatment with a 10 μM concentration of the compounds, cells were harvested, pooled and digested in HNO_3_. The supernatant was used for analysis of Pd(II) and Pt(II) by ICP-MS and the data used to calculate the intracellular Pd-BENSpm, Pd-Spm and Pt-CPENSpm concentrations in MCF-10A (**a**), JIMT-1 (**b**) and L56Br-C1 (**c**) cells. The results are presented as mean values (*n* = 3) and bars represent ± SEM. ***p < 0.001 compared to Pd-Spm or Pt-CPENSpm treatment.  Pd-BENSpm,  Pd-Spm,  Pt-CPENSpm

ICP-MS was used to quantify the Pd(II) or Pt(II) content in cells treated with 10 μM Pd-BENSpm, Pd-Spm or Pt-CPENSpm for 72 h and the data were used to calculate the intracellular concentrations of the drugs (Fig. 2). The data demonstrate that the intracellular concentration of Pd-BENSpm was approximately 20, 40 and 360 pmol/10^6^ cells in MCF-10A (Fig. 2a), JIMT-1 (Fig. 2b) and L56Br-C1 (Fig. 2c) cells, respectively. The Pd-Spm and Pt-CPENSpm concentrations were between 2 and 18 pmol/10^6^ cells in the three cell lines, with the highest concentrations in L56Br-C1 cells and the lowest concentrations in MCF-10A cells.

### Analysis of polyamine levels

**Fig. 3:**
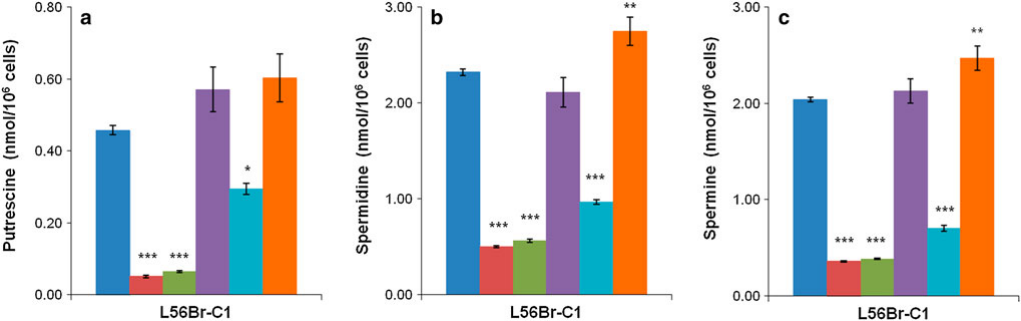
Effect of BENSpm, Pd-BENSpm, Pd-Spm, CPENSpm or Pt-CPENSpm treatment on the polyamine content in L56Br-C1 cells. After 24 h of treatment with a 10 μM concentration of the compounds, cells were harvested, counted in a hemocytometer and then putrescine (**a**), spermidine (**b**) and spermine (**c**) contents were determined by HPLC. The results are presented as mean values (*n* = 3) and bars represent ± SEM. When not visible, the bars are covered by the symbols. *p < 0.05 compared to control; **p < 0.01 compared to control; ***p < 0.001 compared to control.  Control,  BENSpm,  Pd-BENSpm,  Pd-Spm,  CPENSpm,  Pt-CPENSpm

Since L56Br-C1 was found to be the most sensitive cell line, we decided to measure the polyamine levels in these cells after 24 h of treatment with 10 μM of BENSpm, Pd-BENSpm, Pd-Spm, CPENSpm or Pt-CPENSpm (Fig. 3). As expected, the putrescine (Fig. 3a), spermidine (Fig. 3b) and spermine (Fig. 3c) contents decreased significantly upon treatment with BENSpm, Pd-BENSpm or CPENSpm, compared to control. Pt-CPENSpm treatment resulted in a minor increase in polyamine levels compared to control, while Pd-Spm treatment only had a negligible effect (Fig. 3a–c).

### Spermidine/spermine *N*^1^-acetyltransferase activity

Since BENSpm or Pd-BENSpm treatment efficiently depleted the polyamines in L56Br-C1 cells, we decided to measure the activity of the polyamine catabolic enzyme SSAT. The SSAT activity was very low in untreated L56Br-C1 cells, but increased significantly after treatment with BENSpm, Pd-BENSpm or CPENSpm (Table 2). The increase in SSAT activity was twice as high in cells treated with BENSpm or Pd-BENSpm than in CPENSpm-treated cells. SSAT activity was not detected in Pd-Spm- or Pt-CPENSpm-treated cells.

**Table 2:** Spermidine/spermine *N* ^1^-acetyltransferase activity in L56Br-C1cells treated with BENSpm, Pd-BENSpm, Pd-Spm, CPENSpm or Pt-CPENSpm for 24 h

SSAT activity (cpm/10^6^ cells)	
Control	200 ± 39
BENSpm	32,654 ± 4,236
Pd-BENSpm	40,004 ± 5,673
Pd-Spm	n.d.
CPENSpm	15,050 ± 2,390
Pt-CPENSpm	n.d.

After 24 h of treatment with a 10 μM concentration of the compounds, cells were harvested, counted in a hemocytometer and then the spermidine/spermine *N* ^1^-acetyltransferase activity was determined using a radiometric assay. The results are presented as mean values (*n* = 6) and bars represent ± SEM

*n.d.* not detectable

** p < 0.01 compared to control; *** p < 0.001 compared to control

### Cell cycle phase distribution and cell death

Since the various compounds used in the present study were shown to affect cell proliferation differently, we analyzed the effect of BENSpm, Pd-BENSpm, Pd-Spm, CPENSpm or Pt-CPENSpm on cell cycle phase distribution as well as on cell death using flow cytometry (FCM). Cells were sampled for analysis of the cell cycle phase distribution at 24, 48 and 72 h of treatment, but only data for 48 h of treatment are shown in Table 3, since they show the major conclusions.

**Table 3:** Sub-G_1_ region and cell cycle phase distribution of MCF-10A, JIMT-1 and L56Br-C1 cells treated with BENSpm, Pd-BENSpm, Pd-Spm, CPENSpm or Pt-CPENSpm for 48 h

	Control (%)	BENSpm (%)	Pd-BENSpm (%)	Pd-Spm (%)	CPENSpm (%)	Pt-CPENSpm (%)
MCF-10A						
Sub-G_1_	4.3 ± 0.6	1.5 ± 0.1	1.7 ± 0.3	4.6 ± 0.5	3.3 ± 0.2	3.5 ± 0.1
G_1_	73.0 ± 1.1	83.9 ± 0.9	84.9 ± 0.6	63.4 ± 0.9	80.4 ± 0.5	69.3 ± 0.7
S	21.5 ± 0.7	8.0 ± 0.5	7.8 ± 0.4	26.2 ± 0.7	10.5 ± 0.4	22.0 ± 0.8
G_2_	5.5 ± 0.4	8.1 ± 0.4	7.4 ± 0.4	10.4 ± 0.7	9.1 ± 0.6	8.7 ± 0.3
JIMT-1						
Sub-G_1_	2.6 ± 0.3	2.0 ± 0.1	2.2 ± 0.2	2.7 ± 0.1	2.9 ± 0.2	3.0 ± 0.1
G_1_	53.6 ± 1.0	61.2 ± 0.6	62.2 ± 0.8	53.6 ± 0.4	68.3 ± 0.7	67.1 ± 0.3
S	34.4 ± 0.9	29.7 ± 0.1	28.3 ± 0.8	38.0 ± 0.2	23.1 ± 0.7	25.2 ± 0.5
G_2_	12.0 ± 0.1	9.1 ± 0.6	9.5 ± 0.2	8.4 ± 0.2	8.6 ± 0.1	7.7 ± 0.3
L56Br-C1						
Sub-G_1_	28.9 ± 1.6	78.4 ± 0.5	79.7 ± 0.5	29.4 ± 0.5	84.6 ± 0.5	13.3 ± 1.3
G_1_	56.6 ± 1.2	–^a^	–^a^	38.3 ± 2.6	–^a^	53.6 ± 0.7
S	36.4 ± 0.9	–^a^	–^a^	45.6 ± 0.9	–^a^	37.4 ± 0.7
G_2_	7.0 ± 1.3	–^a^	–^a^	16.1 ± 2.2	–^a^	9.0 ± 0.2

Twenty-four hours after seeding the cells, BENSpm, Pd-BENSpm, Pd-Spm, CPENSpm or Pt-CPENSpm was added to a final concentration of 10 μM. At 24, 48 and 72 h of treatment, both detached and attached cells were harvested, pooled and fixed in 70 % ice-cold ethanol. The nuclei were stained with PI and the analysis was performed using flow cytometry. Only results from 48 h of treatment are shown. The results are presented as mean values (*n* = 3) ± SEM. The sub-G_1_ region was evaluated as percentage signals in sub-G_1_ in relation to all signals in the sub-G_1_, G_1_, S and G_2_ histograms. The distribution of cells in the G_1_, S and G_2_ phases was evaluated in % of all cells in G_1_, S and G_2_

^a^In L56Br-C1 cells treated with BENSpm, Pd-BENSpm or CPENSpm, cell death was so prominent that it was not possible to evaluate the distribution of cells in G_1_, S and G_2_ phases

The data show that the only cell line in which the percentage of cells in the sub-G_1_ region, which reflects cell death, increased substantially was the L56Br-C1 cell line (Table 3). In L56Br-C1 cells, cell death was so prominent that is was not possible to evaluate the DNA histograms at 48 and 72 h of treatment. L56Br-C1 cells have a high degree of spontaneous apoptotic cell death, which is obvious in Table 3 where the sub-G_1_ region in control cells is around 20 % (Hegardt et al. 2002). No cell death was observed in MCF-10A and JIMT-1 cells.

In MCF-10A cells, the number of cells in the G_1_ phase increased from around 50 to 80 % between 24 and 48 h of treatment with BENSpm, Pd-BENSpm or CPENSpm (Table 3). During the same time period, the percentage of cells in the S phase decreased from 40 to 10 %. Less evident changes were found in cell cycle phase distribution of JIMT-1 cells after treatment (Table 3).

### Identification of the CD44^+^CD24^−^ putative cancer stem cell population by flow cytometry

**Fig. 4:**
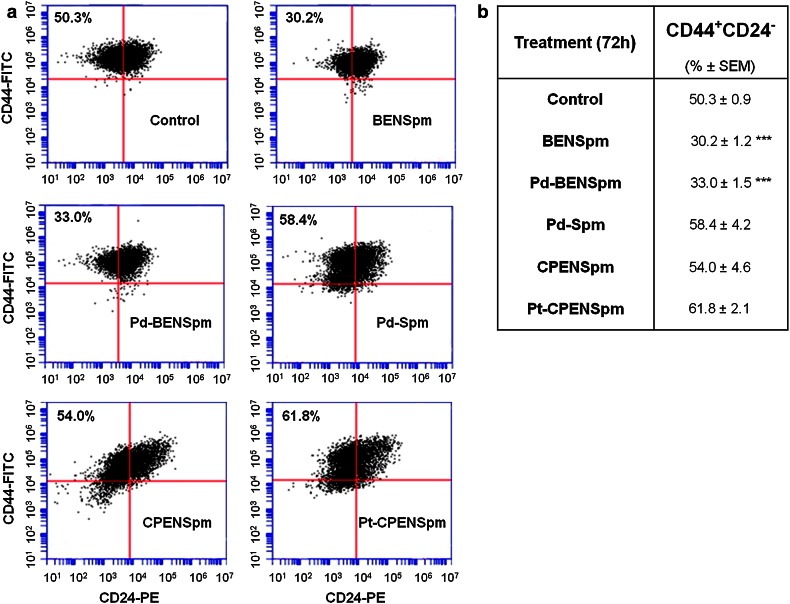
Effect of BENSpm, Pd-BENSpm, Pd-Spm, CPENSpm or Pt-CPENSpm treatment on the CD44^+^CD24^−^ putative cancer stem cell population in JIMT-1 cells. After 72 h of treatment with a 10 μM concentration of the compounds, cells were harvested with Accutase and identified based on their expression of the cell surface markers CD44 and CD24 by flow cytometry. **a** Representative cytograms of the flow cytometric analysis of cell surface-expressed CD44 and CD24 in the JIMT-1 breast cancer cell line. **b** Table showing the data obtained with each treatment. The results are presented as percentage of total population (*n* = 9) ± SEM. ***p < 0.001 compared to control

To investigate the effect on the putative breast cancer stem cell (CSC) population, here defined as CD44^+^CD24^−^ (Al-Hajj et al. 2003; Cirenajwis et al. 2010), JIMT-1 cells were treated for 72 h with 10 μM BENSpm, Pd-BENSpm, Pd-Spm, CPENSpm or Pt-CPENSpm and the cells were analyzed by FCM after labeling with the CD44-FITC and CD24-PE antibodies. As shown in Fig. 4, treatment with either BENSpm or Pd-BENSpm significantly reduced the CD44^+^CD24^−^ subpopulation from 50 to 30 % and 33 %, respectively, whereas treatment with Pd-Spm, CPENSpm and Pt-CPENSpm resulted in a slight increase in the CD44^+^CD24^−^ subpopulation (Fig. 4).

### Colony forming efficiency

The clonogenic assay is designed to measure the ability of single cells to proliferate and form colonies in an anchorage independent manner.

The immortalized normal-like cell line MCF-10A does not form colonies in soft agar and was not used in this assay. In the JIMT-1 and L56Br-C1 breast cancer cell lines, all the treatments decreased the colony forming efficiency (CFE), compared to the control (Table 4). The CFE was similar in JIMT-1 and L56Br-C1 control cells, around 30 %. Pd-BENSpm treatment was most efficient in reducing the number of colonies and L56Br-C1 was the most sensitive cell line to all the treatments (Table 4).

**Table 4:** Effects of BENSpm, Pd-BENSpm, Pd-Spm, CPENSpm or Pt-CPENSpm treatment of JIMT-1 and L56Br-C1 cells on the colony forming efficiency in soft agar

Cell line	JIMT-1	L56Br-C1
Control (%)	29.4 ± 1.0	32.7 ± 1.9
BENSpm (%)	15.1 ± 0.5 (51.2)***	10.8 ± 0.5 (33.0)***
Pd-BENSpm (%)	12.1 ± 0.3 (41.3)***	7.8 ± 0.1 (23.7)***
Pd-Spm (%)	20.5 ± 0.3 (69.7)***	17.0 ± 1.0 (51.8)***
CPENSpm (%)	16.4 ± 0.8 (55.7)***	11.8 ± 1.0 (36.2)***
Pt-CPENSpm (%)	24.1 ± 0.4 (82.1)***	24.0 ± 0.5 (73.2)***

Cells were seeded and the compounds (10 μM) were added 24 h later. After 72 h of treatment, the cells were harvested, counted and reseeded at cloning density in soft agar. Colonies were counted after 14 days of incubation. The results are presented as mean values (*n* = 3) ± SEM and as percentage of control (in brackets)

*** *p* < 0.001 compared to control

### Single cell gel electrophoresis assay

The SCGE assay is a method used to detect DNA damage in single cells and it was used to investigate if the compounds induced DNA strand breaks (Silva et al. 2013).

Table 5 shows the percentage of nucleoids with tails in relation to the total number of nucleoids scored after 72 h of treatment. There were fewer nucleoids with tails in control of MCF-10A and JIMT-1 cells, compared to L56Br-C1 cells. All treatments resulted in an increase in the number of nucleoids with a tail in all cell lines, although the increase was most prominent in L56Br-C1 cells. Of the five tested compounds, Pd-BENSpm was the most efficient in increasing the number of nucleoids with tails. Online Resource 2 shows the comets of L56Br-C1 cells.

**Table 5:** Effects of treatment with BENSpm, Pd-BENSpm, Pd-Spm, CPENSpm or Pt-CPENSpm on the number of comets, i.e., nucleoids with tails in MCF-10A, JIMT-1 and L56Br-C1 cell lines evaluated by the single cell gel electrophoresis assay

Cell line	MCF-10A	JIMT-1	L56Br-C1
Control	4.6 ± 1.0	4.5 ± 1.5	8.1 ± 0.8
BENSpm	11.0 ± 1.6**	18.3 ± 3.7 *	30.5 ± 2.4***
Pd-BENSpm	11.2 ± 1.0**	23.8 ± 4.0 **	41.2 ± 3.8***
Pd-Spm	9.2 ± 0.9	14.3 ± 3.0 *	24.2 ± 2.4**
CPENSpm	11.3 ± 1.5**	16.1 ± 3.9 *	27.2 ± 2.2***
Pt-CPENSpm	9.0 ± 1.6	11.1 ± 2.0	18.4 ± 2.1*

Cells were seeded and the compounds (10 μM) were added 24 h later. After 72 h of treatment, the cells were harvested by trypsinization and the single cell gel electrophoresis assay was performed as previously described. The results show the percentage of nucleoids with tails in relation to the total number of nucleoids scored and are presented as mean values of three independent samples (*n* = 150 cells) ± SEM

* *p* < 0.05 compared to control; ** *p* < 0.01 compared to control; *** *p* < 0.001 compared to control

### GSH-Glo™ Glutathione assay

Since the toxicity of cisplatin has been shown to be dependent on the cellular GSH level (Chen and Kuo 2010), we decided to investigate the GSH level in the cells lines and effects on the level when treating with the compounds.

Table 6 shows that Pd-Spm treatment reduced the GSH level in all cell lines. In fact, in Pd-Spm-treated cells, the level was below the detection limit of the assay and thus the value was set to not detectable (n.d.) Treatment with BENSpm, Pd-BENSpm or CPENSpm resulted in a marked reduction of the cellular GSH levels. The decrease in cellular GSH was most prominent in the L56Br-C1 cells. Pt-CPENSpm treatment, on the other hand, only slightly reduced or even increased the GSH level, depending on the cell line tested.

**Table 6:** Effects of treatment with BENSpm, Pd-BENSpm, Pd-Spm, CPENSpm or Pt-CPENSpm on the levels of glutathione (GSH) in MCF-10A, JIMT-1 and L56Br-C1 cell lines, evaluated by the GSH-Glo™ Glutathione Assay

Cell line	Control	BENSpm	Pd-BENSpm	Pd-Spm	CPENSpm	Pt-CPENSpm
MCF-10A						
GSH (nmol/10^6^cells)	34.3 ± 1.7	25.8 ± 1.8	26.9 ± 2.6	n.d.	22.7 ± 2.5	40.7 ± 2.5
GSH (% of control)		75.1***	78.4***	n.d.	66.2***	118.5**
Cell number (% of control)		55.4	58.8	75.5	52.1	90.4
JIMT-1						
GSH (nmol/10^6^cells)	30.9 ± 1.5	17.1 ± 1.9	7.9 ± 0.8	n.d.	18.6 ± 2.4	20.0 ± 5.8
GSH (% of control)		55.1***	25.6***	n.d.	60.0***	64.8***
Cell number (% of control)		65.9	57.6	70.8	67.7	79.4
L56Br-C1						
GSH (nmol/10^6^cells)	15.1 ± 1.0	2.8 ± 0.4	0.5 ± 0.5	n.d.	1.0 ± 0.2	21.3 ± 4.2
GSH (% of control)		18.6***	3.0***	n.d.	6.8***	140.3***
Cell number (% of control)		19.0	14.9	41.5	20.1	87.0

Cells were seeded in white opaque 96-well plates and the compounds were added 24 h later to a final concentration of 10 μM. After 48 h of treatment, the plates were removed from the incubator and the GSH-Glo™ Glutathione assay was performed according to the instructions of the manufacturer. The results of the GSH assay are presented as nmol/10^6^ cells (mean ± SD) (*n* = 3) and as percentage of control. In Pd-Spm-treated cells, the level was below the detection limit of the assay and thus the value was set to not detectable (n.d.). The data in the row defined as Cell number were derived from the growth curves (Fig. 2a–c). At 48 h of treatment, the number of cells in each treatment was calculated as % of control

** *p* < 0.01 compared to control; *** *p* < 0.001 compared to control

In addition to the effects of the various treatments on the GSH level, Table 6 also shows the effects on cell proliferation (given as % of cells relative to control at 48 h of treatment). The cell proliferation data are derived from the growth curves shown in Fig. 1a–c. Comparing the GSH levels and cell growth, it is clear that there is no obvious correlation between decrease in GSH and reduction of cell number in any of the cell lines.

## Discussion

Cisplatin was the first Pt-based drug used in cancer therapy but, unfortunately, it exhibits severe off-target effects and cancers often acquire resistance to the drug (Lebwohl and Canetta 1998; Wang and Lippard 2005). In an attempt to overcome these limitations, new metal-based antitumor complexes, particularly Pd(II) and Pt(II) polynuclear complexes using polyamines as bridging ligands, have been synthesized over the last decades (Lebwohl and Canetta 1998; Marques et al. 2002; Fiuza et al. 2006, 2011; Soares et al. 2007; Silva et al. 2012).

In the present study, the cytotoxic effects of the metal-based polyamine complexes, Pd-BENSpm, Pd-Spm and Pt-CPENSpm on two breast cancer cell lines (JIMT-1and L56Br-C1) and one immortalized normal-like breast epithelial cell line (MCF-10A) were analyzed. In general, the normal-like MCF-10A cells were less affected by treatment with any of the compounds than the cancer cell lines as shown by the higher IC_50_ value, the lower effects on cell proliferation and on comet formation. In addition, of the very toxic compounds, BENSpm, CPENSpm and Pd-BENSpm, the latter, i.e., Pd-BENSpm, showed the lowest relative toxicity in MCF-10A cells, which is promising.

Previous studies have shown that BENSpm and CPENSpm inhibit cell proliferation of breast cancer cell lines (Holst and Oredsson 2005; Oredsson et al. 2007; Cervelli et al. 2010) and our study confirm those data. In addition, we show that repeated treatment cycles further suppresses cell proliferation. Interestingly, platination of CPENSpm clearly induced a marked reduction in toxicity in all the cell lines analyzed in the present study. In contrast, palladination of BENSpm increased the cytotoxic effect of the compound. One cause for the difference in toxicity may be related to cellular uptake, which was significantly higher for Pd-BENSpm than for Pt-CPENSpm. Earlier studies on Pd(II)- or Pt(II)-NSpd complexes showed that the substitution of Pt(II) for Pd(II) increased the cytotoxicity of the compound (Silva et al. 2013). However, the difference in cytotoxicity between the Pd(II) complex and the Pt(II) complex of NSpd was not explained by a difference in uptake, thus indicating an alternative mechanism. Furthermore, the use of chelating ligands in this fashion may render a very stable Pt(II) compound with reduced lability relative to its parent compound cisplatin and its palladium counterpart and, therefore, less prone to react with cellular targets.

The cell line that was most sensitive to the compounds was L56Br-C1, which was also found for NSpd, Pd-NSpd and Pt-NSpd (Silva et al. 2013). Pd-BENSpm, BENSpm and CPENSpm were all strongly cytotoxic against L56Br-C1 cells, as revealed by growth inhibition, increased cell death, decreased CFE, as well as induction of DNA strand breaks. The uptake of Pd-BENSpm was much more efficient in the L56Br-C1 cells than in the other cell lines tested, which may partly explain the sensitivity of this cell line to the Pd (II) complex. Another possible explanation for the difference in sensitivity may lie in a different basal level of GSH in the cell lines. The GSH pool was about 50 % lower in L56Br-C1 cells compared to that in MCF-10A and JIMT-1 cells. GSH has been shown to be involved in the toxicity of cisplatin. Chen and Kuo (2010) showed that the toxicity of cisplatin was reduced by GSH. Binding of GSH to cisplatin is responsible for metabolic drug inactivation, thereby decreasing its bioavailability at the pharmacological target and hence its antitumor activity (Wu et al. 2013). In addition, cisplatin resistance has sometimes been correlated with a rise in the levels of cellular GSH (Wu et al. 2013).

Interestingly, we found that GSH was not detectable in Pd-Spm-treated MCF-10A, JIMT-1 and L56Br-C1 cells. Pd-Spm was presumably efficiently inactivated by binding to GSH, thus resulting in reduced cytotoxicity. Moreover, Pd(II) is more prone to bind to sulfur atoms than Pt(II) (Lim 1977). Comparing Pd-Spm with Pd-BENSpm, it may be that the latter is more stable and thus the metal is not free to interact with GSH. The fact that the cell number did not decrease after 48 h of treatment with Pd-Spm in any of the cell lines may imply efficient synthesis of GSH, maintaining at least a low pool, although not detectable, of GSH sufficient for cell proliferation and survival. However, this notion has to be further investigated. Treatment with BENSPM, Pd-BENSpm or CPENSpm lowered the GSH pools but not to the same extent as Pd-Spm, implying that these compounds were not inactivated by GSH to the same extent as was Pd-Spm. The Pd-BENSpm chelate thus appears to be more stable that its analogue Pd-Spm, possibly due to the presence of the extra –CH_2_CH_3_ groups at the terminal nitrogens of the polyamine ligand. Moreover, this additional alkylation renders the complex more lipophilic, which can be an advantage for efficiently crossing the cellular and nuclear membranes in its way to the biological target (DNA). Regarding the GSH lowering activity of Pd-Spm, it may be exploited in the search for anticancer redox chemotherapeutics (Wu et al. 2013).

Pd-Spm has previously been demonstrated to be cytotoxic against the breast cancer cell lines MCF-7 and MDA-MB-231 (Fiuza et al. 2011), as well as the human oral squamous carcinoma cell line HSC-3 (Soares et al. 2007). In those studies, the effect of Pd-Spm seemed to be irreversible. No recovery was observed after withdrawal of the drug. Nevertheless, as shown in the present study, the cytotoxic effect of Pd-Spm was, at least, partly reversible in the cell lines JIMT-1 and MCF-10A.

Breast cancer is a heterogeneous disease, composed of tumor cells with different gene expressions and phenotypes (Kao et al. 2009; Kim et al. 2012). The cell surface markers CD44 and CD24 are adhesion molecules and CD44^+^CD24^−^ cells were suggested to be breast cancer stem cells (Al-Hajj et al. 2003). We have previous shown that treatment with the polyamine analogue PG-11047 reduced the putative CD44^+^CD24^−^ CSC population in JIMT-1 cells and decreased their CFE (Cirenajwis et al. 2010). BENSpm, which is a polyamine analogue closely related to PG-11047, also reduced the CSC population evaluated by FCM and the CSC reducing effect was retained to a similar degree by Pd-BENSpm treatment (no significant difference was obtained between BENSpm and Pd-BENSpm). Although the antiproliferative effect of CPENSpm treatment was very much similar to that of BENSpm and Pd-BENSpm treatments, there was no effect on the putative CD44^+^CD24^−^ CSC population. In fact, it increased compared to control. Interestingly, all three compounds resulted in similar decrease in CFE. Thus, the CFE was markedly reduced, whereas the putative CD44^+^CD24^−^ CSC population was not, after treatment with CPENSpm. Moreover, Pt-CPENSpm treatment resulted in an increased putative CD44^+^CD24^−^ CSC population, in spite of a decrease in the CFE, compared to control. Thus, the data support the notion that other factors than only CD44 positivity and CD24 negativity define CSCs (Ricardo et al. 2011). Also, we did the CFE in the presence of FCS, which may support the colony formation by progenitor cells and not only by CSCs. Only CSCs are supposed to form colonies under serum free conditions (Ponti et al. 2005; Fillmore and Kuperwasser 2008), although our notion is that this is not clearly proven.

The generally accepted mechanism of action for this type of metal-based antineoplastic agents is the covalent binding of the metal center [in this case either Pt(II) or Pd(II)] to DNA, particularly to the N_7_ of the purine bases. Thus, a change in structure, and/or in the nature of the metal center, is expected to affect the compound’s efficiency, as observed in this study. Moreover, a change in DNA conformation may also affect the binding of the metal to the DNA. Although, the exact roles of the polyamines are not known it is generally believed that they are important for the DNA conformation. Both BENSpm and CPENSpm have been shown to efficiently deplete cells of their polyamines by down-regulating polyamine biosynthetic enzymes as well as inducing the enzyme catalyzing the initial step in polyamine catabolism, namely SSAT (Davidson et al. 1999; Wolff et al. 2003; Casero and Woster 2009). Interestingly, as shown in the present study, there was a clear correlation between the degree of cytotoxicity and capacity to decrease cellular polyamine levels (and induce SSAT) among the various Pd(II) and Pt(II) polyamine complexes. Thus, it is conceivable that a depletion of polyamines may affect DNA conformation in a way that facilitates the covalent binding of the metal center to the DNA.

A close statistical comparison (not shown) between BENSpm and Pd-BENSpm showed that the latter was indeed somewhat more cytotoxic than the former. The cell number was significantly lower in Pd-BENSpm-treated cultures after 48 and 72 h of treatment than in BENSpm-treated cultures, in all breast cancer cell lines. Pd-BENSpm treatment reduced the CFE significantly more than BENSpm treatment did. Pd-BENSpm treatment resulted in significantly more comets in the SCGE assay than did BENSpm treatment in the two cancer cell lines JIMT-1 and L56Br-C1, but not in MCF-10A cells. Pd-BENSpm treatment also reduced the GSH level significantly more than BENSpm treatment in the two cancer cell lines. Thus, although the difference between BENSpm and Pd-BENSpm is small, Pd-BENSpm showed slightly higher toxicity against cancer cells and thus may be of some importance for further design of new metal-based polyamine analogues.

## Conclusion

In conclusion, the present paper demonstrates that the Pd-BENSpm complex may be regarded as a promising inorganic agent to be used for the development of new chemotherapeutic approaches against breast cancer, due to its slightly higher cancer cell toxicity together with a lower toxicity in the normal-like cell line.

## Electronic supplementary material

Below is the link to the electronic supplementary material.

Supplementary material 1 (TIFF 1,025 kb)

Supplementary material 2 (TIFF 280 kb)

## Abbreviations

BENSpm: *N* ^1^,*N* ^11^-bis(ethyl)norspermine
CFE: Colony forming efficiency
CPENSpm: *N* ^1^-Cyclo-propylmethyl-*N* ^11^-ethylnorspermine
CSC: Cancer stem cell
DMSO: Dimethyl sulphoxide
FCM: Flow cytometry
FCS: Fetal calf serum
FITC: Fluorescein isothiocyanate
GSH: Glutathione
ICP-MS: Inductively-coupled plasma mass spectrometry
MTT: 3-(4,5-Dimethyl-thiazolyl-2)-2,5 diphenyltetrazolium bromide
NSpd: Norspermidine
PBS: Phosphate-buffered saline
Pd: Palladium
Pd-BENSpm: Pd_2_BENSpm
Pd-Spm: Pd_2_Spm
PE: Phycoerythrin
PI: Propidium iodide
polyHEMA: poly(2-hydroxyethyl methacrylate)
Pt: Platinum
Pt-CPENSpm: Pt_2_CPENSpm
SCGE: Single cell gel electrophoresis
SSAT: Spermidine/spermine *N* ^1^-acetyltransferase
