# Supplementary figures and images for: Increased breast cancer cell toxicity by palladination of the polyamine analogue N1,N11-bis(ethyl)norspermine
Source: Amino Acids. 2013 Dec 21;46(2):339–52. doi: 10.1007/s00726-013-1621-y (PMC3906527; doi:10.1007/s00726-013-1621-y)

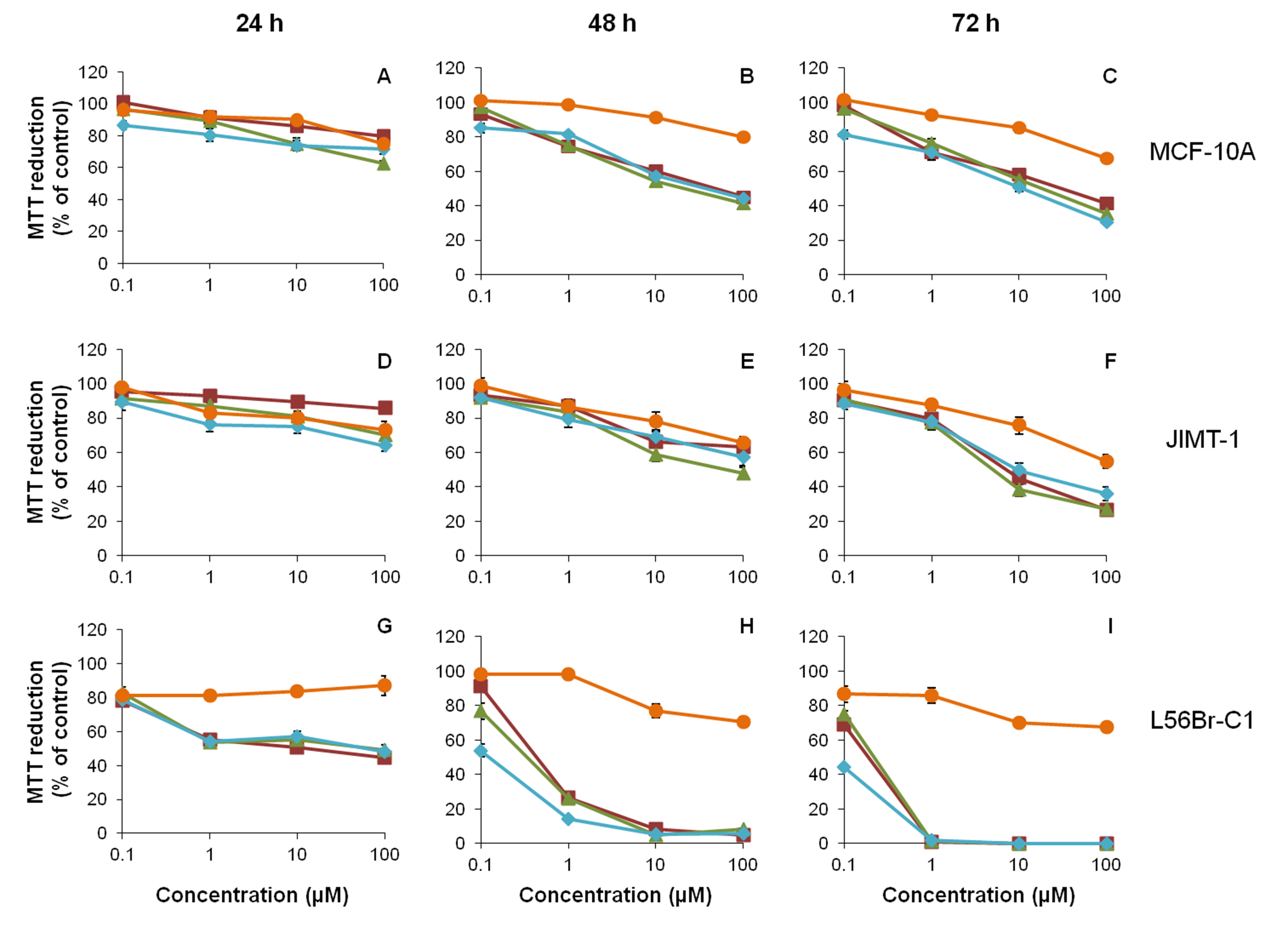

Supplement: Supplementary file 1 — Supplementary material 1 (TIFF 1,025 kb) [file 726_2013_1621_MOESM1_ESM.tif]

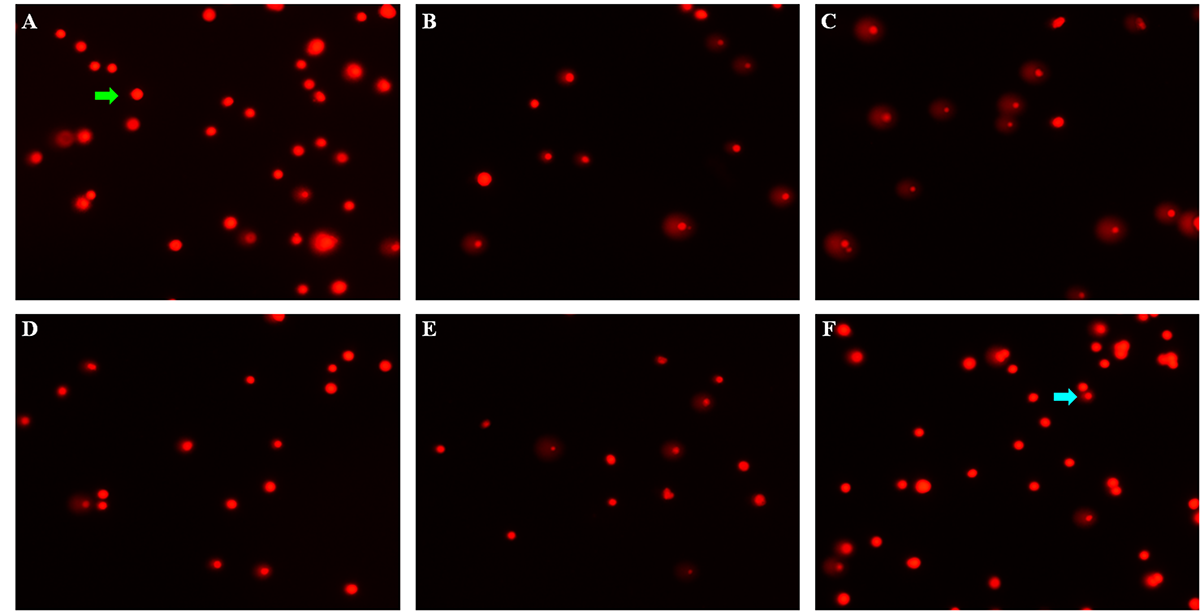

Supplement: Supplementary file 2 — Supplementary material 2 (TIFF 280 kb) [file 726_2013_1621_MOESM2_ESM.tif]
